# Implications of improved representations of plant respiration in a changing climate

**DOI:** 10.1038/s41467-017-01774-z

**Published:** 2017-11-17

**Authors:** Chris Huntingford, Owen K. Atkin, Alberto Martinez-de la Torre, Lina M. Mercado, Mary A. Heskel, Anna B. Harper, Keith J. Bloomfield, Odhran S. O’Sullivan, Peter B. Reich, Kirk R. Wythers, Ethan E. Butler, Ming Chen, Kevin L. Griffin, Patrick Meir, Mark G. Tjoelker, Matthew H. Turnbull, Stephen Sitch, Andy Wiltshire, Yadvinder Malhi

**Affiliations:** 1 Centre for Ecology and Hydrology, Wallingford, Oxfordshire OX10 8BB UK; 2 Division of Plant Sciences, Research School of Biology, The Australian National University, Building 134, Canberra, ACT 2601 Australia; 3 ARC Centre of Excellence in Plant Energy Biology, Research School of Biology, The Australian National University, Building 134, Canberra, ACT 2601 Australia; 4 College of Life and Environmental Sciences, Amory Building, University of Exeter, Rennes Drive, Exeter, EX4 4RJ UK; 5 The Ecosystems Center, Marine Biological Laboratory, 7 MBL Street, Woods Hole, MA 02543 USA; 6 College of Engineering, Mathematics and Physical Sciences, Laver Building, University of Exeter, North Park Road, Exeter, EX4 4QF UK; 7 Department of Forest Resources, University of Minnesota, 1530 Cleveland Avenue North, St Paul, MN 55108 USA; 8 Hawkesbury Institute for the Environment, Western Sydney University, Penrith, NSW 2751 Australia; 9 Department of Earth and Environmental Sciences, Lamont-Doherty Earth Observatory, Columbia University, Palisades, NY 10964-8000 USA; 10 School of Geosciences, University of Edinburgh, Edinburgh, EH9 3FF UK; 11 Centre for Integrative Ecology, School of Biological Sciences, University of Canterbury, Private Bag 4800, Christchurch New Zealand; 12 Met Office, FitzRoy Road, Exeter, Devon EX1 3PB UK; 13 School of Geography and the Environment, Oxford University Centre for the Environment, University of Oxford, South Parks Road, Oxford, OX1 3QY UK

## Abstract

Land-atmosphere exchanges influence atmospheric CO_2_. Emphasis has been on describing photosynthetic CO_2_ uptake, but less on respiration losses. New global datasets describe upper canopy dark respiration (*R*_d_) and temperature dependencies. This allows characterisation of baseline *R*_d_, instantaneous temperature responses and longer-term thermal acclimation effects. Here we show the global implications of these parameterisations with a global gridded land model. This model aggregates *R*_d_ to whole-plant respiration *R*_p_, driven with meteorological forcings spanning uncertainty across climate change models. For pre-industrial estimates, new baseline *R*_d_ increases *R*_p_ and especially in the tropics. Compared to new baseline, revised instantaneous response decreases *R*_p_ for mid-latitudes, while acclimation lowers this for the tropics with increases elsewhere. Under global warming, new *R*_d_ estimates amplify modelled respiration increases, although partially lowered by acclimation. Future measurements will refine how *R*_d_ aggregates to whole-plant respiration. Our analysis suggests *R*_p_ could be around 30% higher than existing estimates.

## Abstract (web summary)

New global datasets of upper canopy vegetation respiration have become available and their impact on global carbon cycle models is unclear. Here, the authors show the implications of these parameterisations with a global gridded land model and report significantly higher global plant respiration estimates.

## Introduction

Fossil fuel burning is increasing atmospheric carbon dioxide (CO_2_) concentrations, which both model- and data-based evidence indicates is warming the planet. Approximately 25% of CO_2_ emissions have been assimilated into terrestrial ecosystems, and whether this continues affects future temperatures. To enable planning for climate change requires robust descriptions of atmospheric CO_2_ capture by photosynthesis (gross primary productivity; GPP) and release by plant (and soil) respiration. The first climate-carbon cycle projection by a global climate model (GCM), HadCM3^1^, identified upper canopy leaf-level dark respiration, *R*_d_ (μmol CO_2_ m^−2^ s^−1^), as a quantity central to predictions of whole-plant respiration. *R*_d_ is parameterised at reference leaf-level temperature 25 °C as *R*_d,25_ (μmol CO_2_ m^−2^ s^−1^). In the HadCM3 simulation^1^, *R*_d,25_ is assumed to be a proportion of maximum carboxylation rate of Rubisco at 25 °C (*V*_cmax,25_ (μmol CO_2_ m^−2^ s^−1^)), itself dependent on mass-based leaf nitrogen concentration, *n*_l_ (kg N (kg C)^−1^). At different leaf-level temperatures, *R*_d_ follows a *Q*_10_ = 2.0 response, thus doubling over 10 °C intervals, although in newer simulations *R*_d_ is suppressed at very high (and low) temperatures^2^ (Methods), and with impact of this assessed for the tropics^3^. Under business-as-usual emissions, by year 2100 in that first simulation^1^, total ecosystem respiratory CO_2_ release overtook GPP, changing the land surface to a source of CO_2_ to the atmosphere. The land surface model in those studies, now named JULES (Joint UK Land Environmental Simulator; Methods and Table 1)^4^, continues operation in Hadley Centre GCMs.

**Table 1:** Standard JULES parameters used and implications for *R*_d,25_ calculation

Variable	Name in JULES model, or derived quantity, (and units)	Broadleaf tree	Needleleaf tree	Shrubs	C_3_ grass	C_4_ grass
$n_{\mathrm{l}0}$	NL0 (kg N (kg C)^−1^)	0.0369	0.0235	0.0349	0.0480	0.0238
$n_{\mathrm{e}}$	NEFFC3 or NEFFC4 (mol CO_2_ m^−2^ s^−1^ kg C (kg N)^−1^)	0.0008	0.0008	0.0008	0.0008	0.0004
*V*_cmax,25_	From parameters above (μmol CO_2_ m^−2^ s^−1^)	36.8	26.4	48.0	58.4	24.0
$f_{\mathrm{dr}}$	FDC3 or FDC4 (Dimensionless)	0.015	0.015	0.015	0.015	0.025
*R*_d,25_	From Eqns above (μmol CO_2_ m^−2^ s^−1^), but before division by the constraints in denominator [(1 + exp(0.3(13.0-*T*_l_))) × (1 + exp(0.3(*T*_l_-36.0)))]	0.4428	0.282	0.4188	0.576	0.238
Final *R*_d,25_	With suppressing constraints from denominator calculated at *T*_l_ = 25.0	0.4157	0.2647	0.3932	0.5407	0.2234

The standard parameters (Methods) used in the JULES model to calculate *R*_d,25_ and for each plant functional type (PFT). However, the *n*_l0_ values use the 50-percentile numbers of the TRY database

Better understanding of plant respiration has become available. Characterisation of *R*_d_ in past studies^1, 4^ was based on the best available *V*_cmax,25_-*n*_l_ and *R*_d,25_-*V*_cmax,25_ parameterisations obtainable at the time (Methods). Recently geographically comprehensive field surveys of *R*_d_ and its temperature dependence have become available, including multi-species comparisons. These new datasets include revised estimates of *R*_d,25_ (GlobResp data)^5^, responses to temperature in the short-term^6^, and longer-term acclimation-type effects^7–9^. Now required is assessment of how these datasets revise models of the global carbon cycle.

The GlobResp dataset^5^ is of upper canopy leaf *R*_d,25_ from ~ 100 sites distributed around the globe, across several biomes and many species (Methods). GlobResp provides *R*_d,25_ parameterisation that scales linearly with leaf nitrogen concentration, *n*_l,a_ (gN (m^2^ leaf)^−1^), via parameter *r*_1_ (μmol CO_2_ m^−2^ s^−1^ (gN (m^2^ leaf)^−1^)^−1^) (along with offset parameter *r*_0_ (μmol CO_2_ m^−2^ s^−1^)), and in a plant functional type (PFT)-dependent manner. Higher *n*_l,a_ values increase *R*_d__,25_. A recent compilation^6^ of 673 high-temporal resolution, short-term instantaneous responses of leaf *R*_d_ to upper-canopy leaf temperature *T*_l_ (°C), again from across the globe (Methods), show convergence in leaf *R*_d_-*T*_l_ response across biomes and PFTs. Analysis of the dataset^6^ reveals a single empirical response curve, as an exponential-of-quadratic, fits well and with two coefficients *b* and *c* that gives an effective *Q*_10_ continuously declining with increasing *T*_l_. This is different from earlier observations^10^ and models^11, 12^. Across the range of leaf temperatures in nature, application of this response^6^ does not predict a decrease in *R*_d_ for high *T*_l_; we refer to this short-term response as the *b,c* temperature formulation.

The GlobResp dataset^5^ additionally shows leaf *R*_d,25_ as highest at cold high latitudes, and lowest in warm tropical environments, consistent with acclimation adjustments when plants experience sustained differences in growth temperature^7–9^. Recent modelling studies^13, 14^ include thermal acclimation of GPP (via shifts in temperatures at which peak rates of carboxylation (*V*_cmax_) and electron transport (*J*_max_) occur^15^), and *R*_d_ via mean air temperature of preceding 10 days^16^. The latter study^16^ uses data on *R*_d_ from juveniles of 19 plant species grown under hydroponic and controlled environment conditions^17^; GlobResp, however, is a dataset roughly 50 times larger and based on mature plants in the field across global climate gradients. Retaining that respiration acclimates to mean air temperature of the preceding 10 days^17^ (*T*_G_ (°C)), GlobResp implies the most robust procedure to account for thermal acclimation is a linear, *T*_G_-dependent perturbation of *R*_d__,__25_ (through parameter *r*_2_), decreasing by 0.0402 μmol CO_2_ m^−2^ s^−1^ °C^−1^ as *T*_G_ increases. As timescales down to just 10 days influence *R*_d,25_, then by some definitions this acclimation includes, implicitly, longer-term evolutionary adaptation effects.

The combination of *R*_d,25_ = *r*_0 +_ *r*_1_ *n*_l,a_−*r*_2_ *T*_G_ description^5^ and *b, c* formulation^6^ for *T*_l_, gives upper-canopy leaf-level respiration as:

1 $$R_{\mathrm{d}} = \left[ {r_o + r_1n_{{\mathrm{l}},{\mathrm{a}}} - r_2T_{\mathrm{G}}} \right] \times \,e^{\left[ {b\left( {T_{\mathrm{l}} - 25} \right) + c\left( {T_{\mathrm{l}}^2 - 25^2} \right)} \right]}$$

with values^6^ of *b* = 0.1012 °C^−1^ and *c* = −0.0005 °C^−2^. We now implement this description of *R*_d_ in to the JULES large-scale land model^4^. Linear mixed-effects models for the GlobResp dataset show for four PFTs presently in the JULES model, particular parameters (Table 2) capture much species variation across diverse sites. PFT-dependent *n*_l,a_ are from the TRY database^18^. Our overall finding is that assimilating the comprehensive GlobResp dataset with the JULES terrestrial ecosystem model yields plant respiration rates that are considerably larger than current estimates. The relative importance of contributions (Methods) to revised *R*_d,25_ values are broad changes to overall baseline having most influence (via parameters *r*_0_, *r*_1_ and *r*_2_ considered together), followed by the specific acclimation dependency and then the relationship with leaf nitrogen.

**Table 2:** Parameter values used in Equation 1

Regression coefficient (and units)	Broadleaf trees (BT)	Needleleaf trees (NT)	Shrubs (S)	C_3_ grasses	C_4_ grasses
*r*_0_ (μmol CO_2_ m^−2^ s^−1^)	1.7560 ± 0.2180	1.4995 ± 0.1793	2.0749 ± 0.0774	2.1956 ± 0.1408	n/a
*r*_1_ (μmol CO_2_ m^−2^ s^−1^ (gN (m^2^ leaf)^−1^) ^−1^)	0.2061 ± 0.0023	0.2061 ± 0.0023	0.2061 ± 0.0023	0.2061 ± 0.0023	n/a
*r*_2_ (μmol CO_2_ m^−2^ s^−1^ (^o^C) ^−1^)	0.0402 ± 0.0096	0.0402 ± 0.0096	0.0402 ± 0.0096	0.0402 ± 0.0096	n/a
*n*_l0_ (kg N (kg C)^−1^)	0.0369	0.0235	0.0349	0.0480	0.0238
$\sigma _{\mathrm{l}}$ (kg C (m^2^ leaf)^−1^)	0.0506	0.112	0.0512	0.0248	0.0656
*n*_l,a_ (gN (m^2^ leaf)^−1^) from *n*_l,0_ x $\sigma _{\mathrm{l}}$ × 10^3^	1.867	2.632	1.787	1.190	n/a
*R*_d,25_ with *T*_G_ = 25 ^o^C (μmol CO_2_ m^−2^ s^−1^)	1.136	1.037	1.438	1.436	n/a

Parameters *r*_0_, *r*_1_ and *r*_2_, which define *R*_d,25_^5^ and for each plant functional type (PFT). Intermediate rows provide values for calculation of *n*_l,a_. Values of nitrogen content prescribed to Joint UK Land Environmental Simulator (JULES) (*n*_l0_) and specific leaf density (*σ*_l_), used to calculate nitrogen content in area-based units, as *n*_l,a_, are the 50-percentiles across the TRY database for the PFTs. The last row shows values of *R*_d,25_ calculated using Eq. (1), assuming *T*_l_ = *T*_G_ = 25 °C. The GlobResp database contains global patterns in upper canopy leaf-level respiration, *R*_d_, of BTs, NTs, Ss and C_3_ grasses. Comparable data for C_4_ grasses remains lacking (n/a), and hence standard JULES values for *R*_d_ are used. Uncertainty bounds are ± one standard errors

## Results

### Numerical simulations

**Fig. 1:**
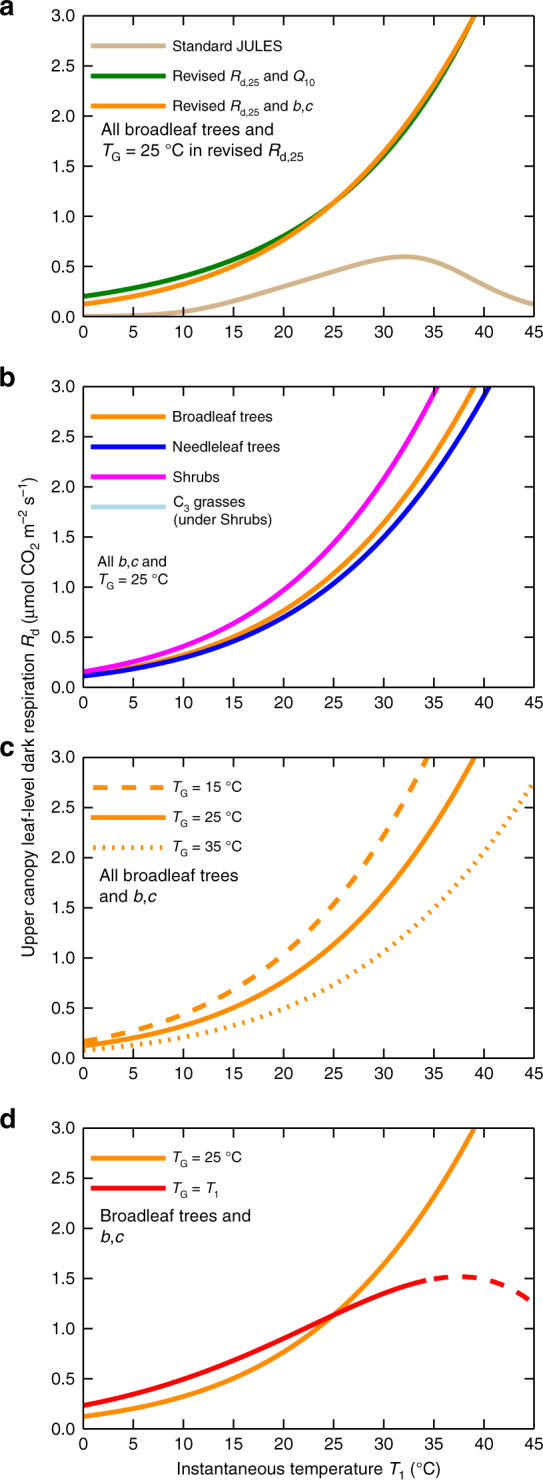
Upper canopy leaf-level dark respiration. **a** Standard Joint UK Land Environmental Simulator (JULES) model (with TRY-based *σ*_l_ and *n*_l0_ values and *Q*_10_ response modulated at high and low temperatures (Methods)). Also revised *R*_d,25_ (*T*_G_ = 25 °C) with both *Q*_10_ = 2.0 and *b,c* responses to *T*_l_. **b** New *R*_d,25_ and *b,c* response to *T*_l_, for other PFTs. C_3_ grasses near identical curve to Shrubs. **c** New *R*_d,25_ and *b,c* formulation for broadleaf trees, but alternative acclimation temperatures. **d** New *R*_d,25_ and *T*_l_ as *b,c* again broadleaf trees, for both *T*_G_ = 25 °C and *T*_G_ = *T*_l_. Orange curve common all panels. Light inhibition not included in responses

Figure 1 presents implications of new *R*_d_ components of Eq. (1). Figure 1a shows for broadleaf trees significant increases across all temperatures in respiration compared to standard JULES, when using the new *R*_d,25_ values (*T*_G_ = 25 °C) and either *Q*_10_ = 2 or the *b,c T*_l_ response. Figure 1b shows the four PFT responses to *T*_l_, with revised *R*_d,25_ values, *T*_G_ again 25 °C, and *b*,*c* formulation. Figure 1c illustrates strong *R*_d_ differences of Eq. (1) between acclimation temperatures *T*_G_ = 15, 25 and 35 °C (for broadleaf trees). In Fig. 1d, the orange curve is the same *R*_d_−*T*_l_ response (*T*_G_ = 25 °C) as in Fig. 1c. However, the red curve sets acclimation temperature equal to instantaneous temperature i.e. *T*_G_ = *T*_l_. This sensitivity test recognises that although acclimation growth temperature, *T*_G_, is determined over longer 10 day periods, higher *T*_G_ values will be geographically where *T*_l_ is higher and vice versa. This dampens *R*_d_ variation in *T*_l_. Curve dashed for extremely rare temperatures *T*_G_ > 35 °C.

JULES scales *R*_d_ to canopy-level respiration, *R*_d,c_ (μmol CO_2_ m^−2^ s^−1^). It can calculate CO_2_ exchange at each canopy level^19^, including dependence on vertical decline of leaf nitrogen^19^ and differentiation of direct and diffuse radiation^20^. However, data are unavailable for how well Eq. (1) performs at lower canopy levels, even if nitrogen concentration and temperatures are known. Given this, we use a simple big-leaf exponential decline in leaf respiration throughout the canopy, decay co-efficient *k* = 0.5 and dependent on leaf area index (LAI). Implicit is that canopy nitrogen and light levels decay identically, affecting respiration and photosynthesis. A 30% light inhibition of non-photorespiratory mitochondrial CO_2_ release^21^ is included for light above ~ 2 W m^−2^. *R*_d,c_ is also reduced by any soil moisture stress^4^. Other respiratory components^4^ include maintenance respiration of stems and roots. These are modelled as larger for canopies with high LAI, higher nitrogen concentrations and also described as scaled in *R*_d,c_. Combining stem and root respiration with *R*_d,c_ gives whole-plant maintenance respiration, *R*_pm_ (Methods). JULES calculates growth respiration *R*_pg_ as linearly increasing (via co-efficient *r*_g_) with plant GPP minus *R*_pm_. Combining *R*_pm_ and *R*_pg_ gives whole-plant respiration, *R*_p_ (μmol CO_2_ m^−2^ s^−1^). Hence changes to *R*_d_ description influence all respiration components that add together to give *R*_p_.

**Fig. 2:**
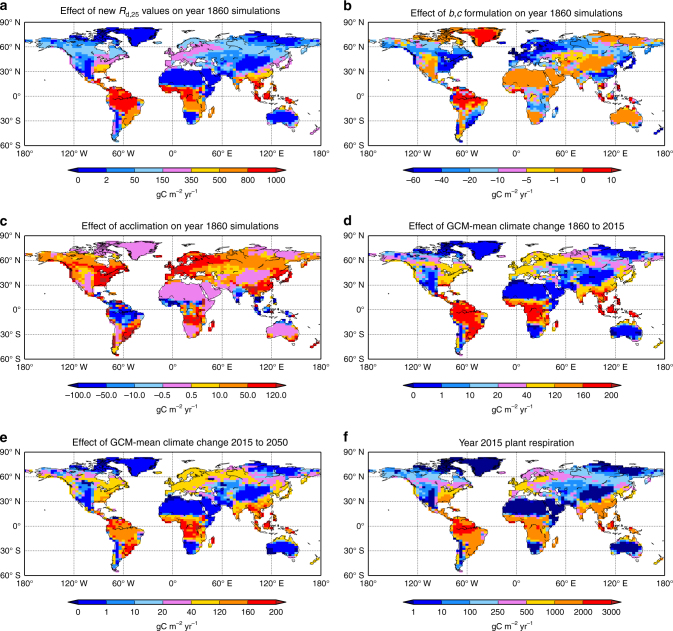
Gridbox-mean maps of total plant respiration for new processes and imposed climate change. Changes to *R*_p_ as: **a** introduction of new *R*_d,25_ values: New_*R*_d,25_ minus Standard; **b** effect of new instantaneous *T*_l_ response: New_*R*_d,25__*b,c* minus New_*R*_d,25_; **c** effect of acclimation: New_*R*_d,25__*b,c*_acclim minus New_*R*_d,25__*b,c*; **d** effect of climate change to present, as year 2015 minus year 1860 and new processes New_*R*_d,25__*b,c*_acclim; **e** similar to **d**, but year 2050 minus year 2015. Panel **f** actual 2015 values of New_*R*_d,25__*b,c*_acclim. Scales different between panels to highlight effects. Units are SI. Panels **d**–**f** are means across the 34 GCMs emulated

Spatial gridded calculations enable geographical implications of revised *R*_d_ description to be determined. JULES is first forced by meteorological conditions near to pre-industrial state, using UK Climate Research Unit data and atmospheric CO_2_ concentration of 280 ppm. To understand the implications of each part of our new *R*_d_ description and as given by Eqn. (1), we add sequentially each component to understand its relative importance. Four sets of simulations are as follows. The first is called Standard—this is standard default JULES (although with TRY-based *n*_l0_ and *σ*_l_ values, as in all simulations; *Q*_10_ response but high/low temperature suppression—see Methods). The second is called New_*R*_d,25_—this is new baseline alone, hence new *R*_d,25_ values^5^, plus *Q*_10_ = 2.0 (no suppression) *T*_l_ response and fixed *T*_G_ = 25 °C. The third is called New*_R*_d,25_ *_b,c* —this is new baseline and instantaneous response i.e. new *R*_d,25_ values and *T*_l_ response with *b,c* formulation, but still *T*_G_ = 25 °C. The fourth is called New*_R*_d,25_ *_b,c_*acclim—this is all factors including acclimation, i.e., new *R*_d,25_ values, *b,c* formulation and acclimation via variation in *T*_G_. The fourth simulations are therefore for the full Eq. (1)^5, 6^. Figure 2a–c shows how each of these new components of our respiration function uniquely influences whole-plant respiration globally for pre-industrial climate forcings. Figure 2a shows annual gridbox mean *R*_p_ (weighting PFTs by fractional cover) for New*_R*_d,25_ minus Standard simulations. This shows that altered baseline through the new *R*_d,25_ (and removed suppression) causes large *R*_p_ increases, including at mid-Northern latitudes and especially for the tropics. Figure 2b shows New*_R*_d,25_ *_b,c* minus New*_R*_d,25_, illustrating the implications of the new instantaneous temperature description. The *b,c* formulation suppresses respiration in mid-latitudes but enhances for the tropics, although changes are smaller than Fig. 2a. Figure 2c presents New*_R*_d,25_ *_b,c_*acclim minus New*_R*_d,25_ *_b,c*, showing acclimation introduction generally increases predicted pre-industrial *R*_p_, except in the tropics where acclimation to higher temperatures lowers respiration.

To estimate anthropogenically-induced climate change, changes in near-surface meteorological conditions use the Integrated Model Of Global Effects of climatic aNomalies (IMOGEN) pattern-scaling system^22, 23^ (Methods) responding to altered atmospheric greenhouse gas (GHG) concentrations. Patterns are calibrated against 34 GCMs of the Coupled Model Intercomparison Project Phase 5 (CMIP5)^24^, with identical method to that originally undertaken for the HadCM3 GCM^22^. We use known historical, then future GHG concentrations of the RCP8.5 business-as-usual representative concentration pathway^25^. Figure 2d shows, for the most complete updated model, New*_R*_d,25_ *_b,c_*acclim, that historical climate change increases *R*_p_ in most locations and especially tropics, despite acclimation dampening stimulatory warming effects. Figure 2e presents calculations between years 2015 and 2050, showing *R*_d_ with similar changes to recent past in Fig. 2d. Figure 2f presents year 2015 absolute *R*_p_ values for New*_R*_d,25_ *_b,c_*acclim case.

**Fig. 3:**
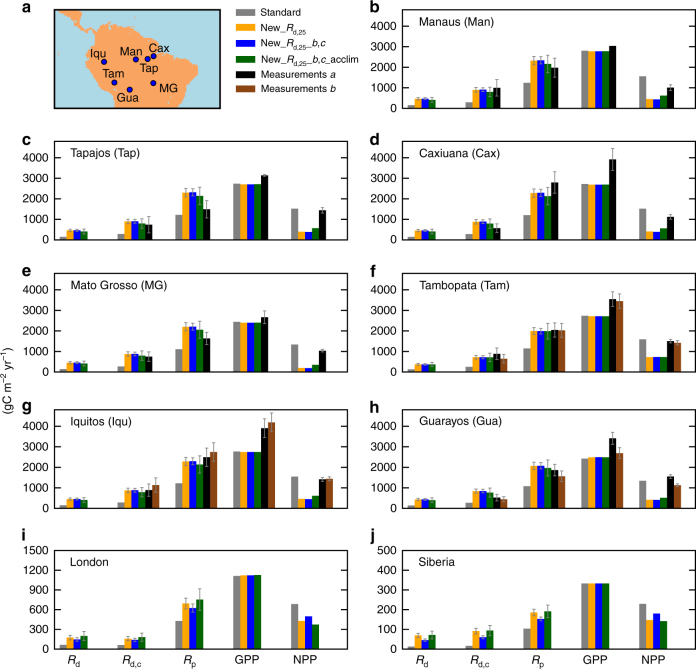
Respiration and primary productivities (gross as GPP and net as NPP) at selected locations during modelled year 2015. Seven locations (details in Methods) **a** are South American **b**–**h**, along with **i** for gridbox containing London, UK, and **j** is in Siberia, Russia (Lat 70 N, Lon 82.5 E). Shown for dominant plant functional type (PFT) at each site, left to right, for each histogram cluster: upper canopy leaf-level respiration (with light inhibition) *R*_d_, whole canopy-level respiration *R*_d,c_, total plant respiration *R*_p_, GPP and NPP. Each histogram cluster are four estimates: Standard, New_*R*_d,25_, New*_R*_d,25_ *_b,c* and New*_R*_d,25_ *_b,c_*acclim. South America sites, 5th (or 6th) column are measurements. Dominant PFTs: **b**–**h** BTs, **i** grasses, **j** shrubs. Uncertainty bounds of ± one s.d. are presented which for model estimates are from data-based upper canopy leaf-level uncertainty estimates, subsequently propagated through the model. For measurements, these bounds are taken from the literature

Figure 3 presents model output for single illustrative locations and in year 2015. For our four simulations, presented are respiration components (*R*_d_, *R*_d,c_ and *R*_p_), plus GPP and NPP. We chose seven sites across South America, a temperate grassland (London) and boreal region shrubs (Siberia). We select multiple South America sites (Methods), as these are some of the few where measurements are available of all respiration components. In general, new *R*_d,25_ values, whether also with or without adjustment by the *b,c* formulation and acclimation, give marked increases in predicted respiration. Transition to whole canopy (*R*_d,c_) and whole plant (*R*_p_) respiration illustrates how our leaf level changes propagate to these aggregated fluxes. Uncertainty bounds^5^ on *r*_0_, *r*_1_ and *r*_2_ are propagated through the JULES model (Methods) to give uncertainty on *R*_d,c_ and *R*_p_ as shown in Fig. 3, while measurement uncertainty is from the literature describing each site. For South American sites, and with our choice of big-leaf approximation, our changes reproduce whole-canopy respiration *R*_d,c_ better (i.e., model and data uncertainty bounds overlap, and better than the default Standard JULES configuration), and in some instances also *R*_p_. More specifically, we define the JULES model as having improved performance when the Standard simulation estimate of *R*_p_ lies outside the data-based bounds on whole-plant respiration, but simulations New*_R*_d,25_ *_b,c_*acclim then fall within those bounds. This happens for the sites at Manaus, Tambopata, Iquitos (dataset a), and Guarayos (dataset a). However, when subtracting *R*_p_ from GPP estimates, NPP values are generally too small. We note that observations of nitrogen at different canopy positions from tropical tree species suggest an effective decay co-efficient *k* with value nearer to 0.2 than 0.5^26^. Using this to scale, and with Eqn (1) still used for upper canopy levels, gives exceptionally large *R*_p_ values and unsustainable negative NPP.

**Fig. 4:**
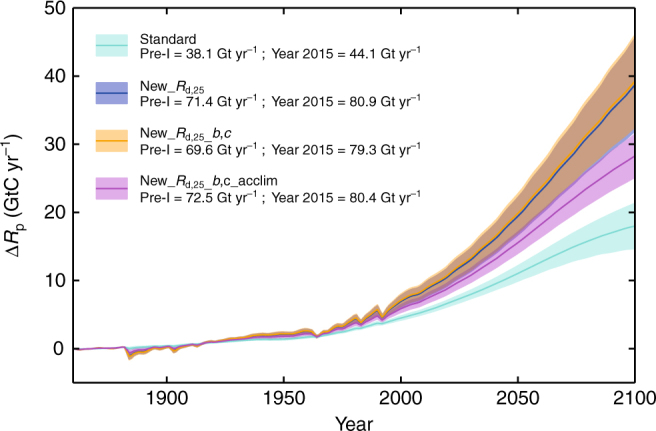
Time series of change in areally-averaged global respiration. Presented are time-evolving model estimates of change in total whole-plant respiration, Δ*R*_p_. The colours of turquoise, blue, yellow and magenta are Standard, New_*R*_d,25_, New_*R*_d,25__*b,c* and New_*R*_d,25__*b,c*_acclim respectively. Where yellow and blue projections overlap, the colour is brown. The spread corresponds to the different projections of climate drivers, based on the 34 Global Circulation Models (GCMs) emulated in the Integrated Model Of Global Effects of climatic aNomalies (IMOGEN) modelling system and for RCP8.5 scenario. The continuous lines are the mean, and the spread as ±  two s.d. which broadly covers inter-GCM spread. Pre-industrial (marked Pre-I) and year 2015 model mean absolute estimates of *R*_p_ are as annotations

Figure 4 shows global time-evolving changes, since pre-industrial times, in total whole-plant respiration, Δ*R*_p_ (GtC yr^−1^) and for our four RCP8.5 scenario simulations. Annotated are pre-industrial and 2015 absolute *R*_p_ estimates. Replacement of standard JULES with GlobResp-based^5^ *R*_d,25_ values (still *Q*_10_ = 2, although with no high or low temperature suppression) approximately doubles both pre-industrial respiration estimates (as marked) and the projected changes in Δ*R*_p_ under climate change. Replacing *Q*_10_ with *b,c* formulation^6^ causes slight global changes. Thermal acclimation increases *R*_p_ slightly for pre-industrial but decreases evolving Δ*R*_p_, i.e., comparing simulations New*_R*_d,25_ *_b,c_*acclim and New*_R*_d,25__*b,c*. The stimulatory effect of acclimation arises from the higher predicted rates in globally widespread biomes where *T*_g_ < 25 °C, but then dampens responses of such sites to future warming. Our new global *R*_p_ values (80.4 GtC yr^−1^ in 2015 for New*_R*_d,25_ *_b,c_*acclim simulations) are higher than other estimates for contemporary periods. One^27^ global GPP estimate is 119.6 GtC yr^−1^, and balancing soil plus plant respirations will be similar magnitude i.e. together they are also of order 120 GtC yr^−1^. With soil respiration equivalent in size to *R*_p_, this suggests plant respiration of order 60 GtC yr^−1^. Our analysis implies global *R*_p_ values could be ~30% higher than that value. However, this estimate is not just a consequence of the entrainment of GlobResp data in to the JULES model, but also the scalings within it and as illustrated at selected geographical points in Fig. 3.

The GlobResp database^5^ is sufficiently comprehensive as to be globally representative for our simulations. Our analysis has implications for other land ecosystem modelling groups. From a survey of ten leading land surface models, six of these simulate leaf respiration with a dependency on nitrogen content (models listed in Methods). In addition as is common to most land surface carbon cycle models (e.g., also for the Lund Potsdam Jena LPJ model (Table 1 of ref. ^28^)), the JULES system calculates maintenance respiration for other components of roots and stems that are based on their carbon and estimated nitrogen content. This approach follows pioneering research^29^ which moved respiration representation on from simply a fraction of GPP, as had been assumed beforehand. We expect the impact of changing the temperature function itself to at least be common among the current generation of models. However this does open research questions as to how Eq. (1) might change at lower positions in canopies, and whether root, stem and growth respiration models require refinement. This is especially because our GlobResp-based changes propagate directly through modelled canopies by JULES (Eqs. 42–25 in ref. ^4^). Hence higher upper-canopy *R*_d_ values then generate larger rates of whole-plant respiration, *R*_p_, than other estimates.

**Fig. 5:**
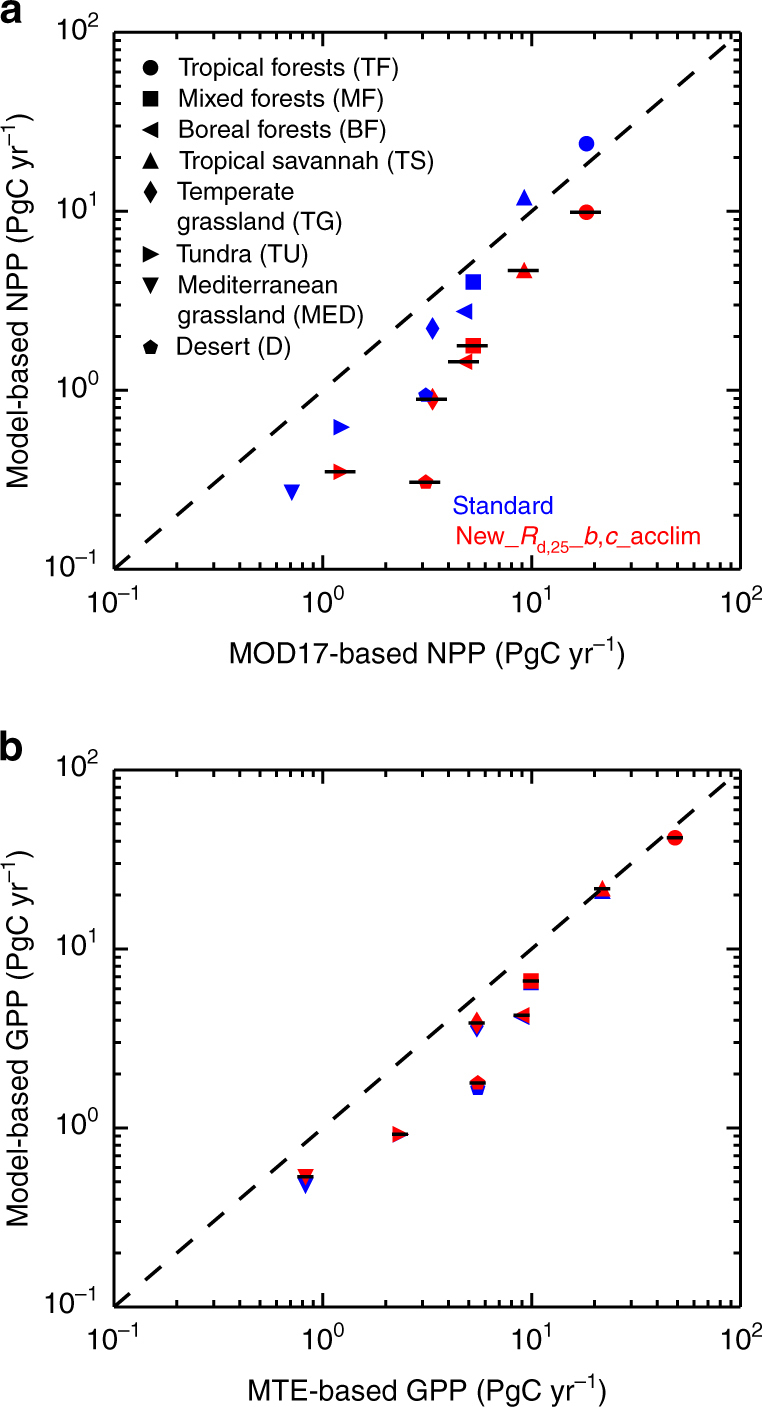
Data- and model-based global estimates of net primary productivity and gross primary productivity for different biomes. **a** Global measurements of total annual mean net primary productivity (NPP), average for years 2000–2011, and using Earth-observed MODerate-resolution Imaging Spectroradiometer (MODIS) measurements. Values are spatially aggregated for different World Wildlife Fund (WWF) biome classifications. The dominant biome type at each location is linked to NPP with the MOD17 algorithm applied to MODIS values (horizontal axis). Similarly gridbox-mean JULES estimates of NPP are multiplied by gridbox area, and combined for each WWF biome (vertical axis). This is dependent upon which WWF biome is dominant for the grid location. Note logarithmic axes. JULES NPP estimates are slightly negative for Mediterranean grasslands and so off axes. **b** Similar calculation for gross primary productivity (GPP), with measurements from the Model Tree Ensemble (MTE) algorithm. Both panels, model values presented in blue for standard JULES version (i.e., Standard simulation), and in red for new *R*_d,25_ values with *b,c* temperature response and acclimation (i.e., New*_R*_d,25_ *_b,c_*acclim simulation). For GPP, differences are small between two model forms, with red symbols overlapping blue symbols. Uncertainty bounds on NPP and GPP data are the small black horizontal bars ( ± one s.d.), shown for red symbols only. All calculations include only land points with less than 50% agriculture

Benchmarking tests of modelled respiration fluxes will be important. For instance, the International LAnd Model Benchmarking project (ILAMB)^30^ is a comprehensive system collating datasets relevant to land surface functioning and of importance to land surface respiration is the Global Bio-Atmosphere Flux (GBAF)^31^ dataset based on extrapolation of eddy-covariance FLUXNET sites. Also available are estimates of global soil respiration^32^, which in conjunction with GBAF measurements can return total plant respiration, at least for comparison at night-time periods. Presently, however, without comprehensive measurements of other canopy components, it is difficult to attribute any discrepancies to GlobResp versus lower-canopy, stem, root or growth contributions. Should higher *R*_p_ values imply especially low values of NPP, then GPP parameterisation may need reassessment; other analyses suggest current estimates of GPP may be too low^33^. Despite this, in Fig. 5 we perform large-scale comparisons against two Earth Observation-based datasets. These are estimates of NPP from the MODerate-resolution Imaging Spectroradiometer (MODIS) satellite, using the MOD17 algorithm^34, 35^, and of GPP from the Model Tree Ensemble (MTE) method^27^. For both datasets, we evaluate mean NPP and GPP values depending on location, and mapping these on to local dominant biomes based on the World Wildlife Fund (WWF) ecoregion classifications^36^ (Methods). These data-based estimates locally represent mean NPP and GPP, and so for parity we compare against modelled gridbox mean JULES calculations of the equivalent quantities. That is, we use areal weighting of the five PFT types in JULES for each position. To keep similarities with the WWF categories, we plot in Fig. 5 total annual NPP and GPP for both data and JULES, integrated over areas for the named biomes as marked. Presented are Standard and New*_R*_d,25_ *_b,c_*acclim simulations. Calculations with New*_R*_d,25_ and New*_R*_d,25_ *_b,c* model format are very similar to New*_R*_d,25_ *_b,c_*acclim and so not shown. As expected, in all cases, introduction of GlobResp-based respiration estimates results in much lower modelled NPP values. Furthermore for New*_R*_d,25_ *_b,c_*acclim simulations and all eight biomes, these are significantly less than MODIS-based measurements. The two sets of simulations have similar GPP estimates, illustrating weak indirect couplings in the JULES model between respiration changes and influence (e.g., via hydrological cycle) on gross primary productivity. It is noted in Fig. 5b that JULES model estimates of GPP are similar to the MTE-based data for tropical forests and tropical savannahs. Uncertainty bounds on data adopt the global literature values of ± 15% for NPP^37^ and ± 7% for GPP^38^. These are the small horizontal black bars, shown only on New*_R*_d,25_ *_b,c_*acclim red points.

**Fig. 6:**
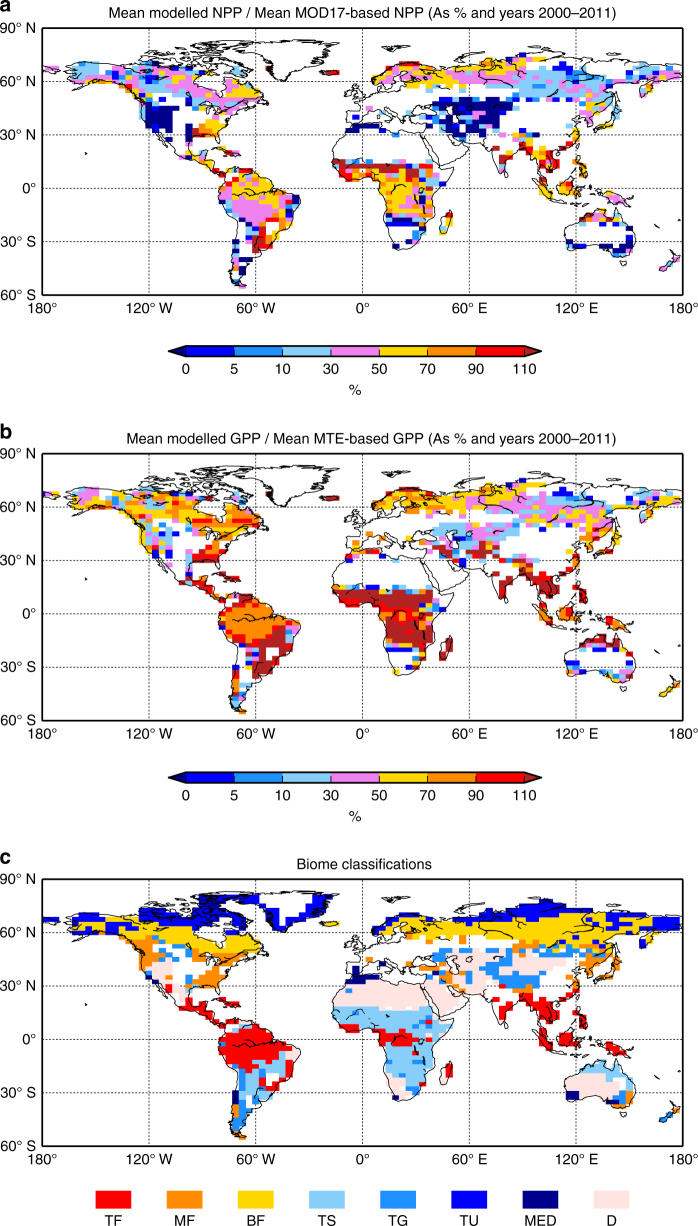
Data- and model-based maps of comparison of net primary productivity and gross primary productivity for different biomes. **a** Map of JULES estimates of annual NPP, average for year 2000-2011 divided by MODIS NPP algorithm (MOD17) estimates for the same period. Values multiplied by one hundred to express as percentage. Land points excluded are those with >50% agriculture, and also where values are very small (if absolute value of JULES or MODIS NPP is less than 1 gC m^−2^ yr^−1^). **b** Similar to (**a**) but for GPP, and data based on upscaled FLUXNET GPP from the MTE algorithm. Again, land points excluded are those with > 50% agriculture, and those with small values (if value of JULES or MTE-based GPP is less than 1 gC m^−2^ yr^−1^). Panel **c** is map of dominant biome, and labels identical to Fig. 5

In Fig. 6, we add geographical information to our global data estimates of NPP and GPP, and for corresponding JULES simulations with all effects, i.e., New*_R*_d,25_ *_b,c_*acclim (expanding on the red symbols of Fig. 5). Figure 6a is JULES NPP estimates divided by MOD17-based NPP estimates (and multiplied by 100 to give percentage). In general modelled NPP with new plant respiration description, is smaller than MOD17 NPP across the geographical points. For some points it can give unsustainable negative modelled NPP values. For GPP, the situation is slightly less clear. Figure 6b is JULES GPP estimates divided by MTE-based GPP values, again as percentage. For many points, the JULES model is also underestimating GPP, and this includes much of the Amazon region. However, for the tropics, a few modelled GPP values are actually higher than data. This offers an explanation as to why GPP appears underestimated in some tropical points of Fig. 3, yet for the average across Tropical Forest (TF), JULES performs well (Fig. 5b). Figure 6b also shows that modelled GPP is usually too low outside of the tropics. This is why, when combined with the enhanced respiration of New*_R*_d,25_ *_b,c_*acclim formulation, this can lead to very low or even unsustainable negative NPP. Figure 6c shows the dominant WWF-defined biomes for each location.

## Discussion

Inversion studies suggest roughly 25% of CO_2_ emissions are presently assimilated by the land surface^39^. Hence net ecosystem productivity (NEP) is ~ 2.5 GtC yr^−1^, implying a small difference between GPP and total ecosystem respiration (whole-plant plus soil) fluxes. Here we have entrained the GlobResp dataset^5^ of upper-canopy respiration with a well-established land surface model JULES which aggregates *R*_d_ through to whole-plant respiration. This implies higher whole-plant respiration, and therefore may need to be balanced by either higher GPP values^33^ or the multiplicative dependence of other components on *R*_d_ is too large. As global land-atmosphere CO_2_ fluxes are a small difference between large fluxes, future terrestrial ecosystem respiration responses to warming can therefore influence the natural ability to offset CO_2_ emissions. This is particularly important as land warmings are projected to be higher than global mean rise^40^. The recent pause in growth rate of atmospheric carbon dioxide has been linked to the warming hiatus suppressing respiration whilst CO_2_ fertilisation continues^41^. If future increases in respiration overtake any thermal or CO_2_-ecosystem fertilisation, lower NPP values in the most extreme instances could force biome changes^1^; this will require operation of the interactive vegetation component of land surface models to assess (Methods). Equivalent global respiration measurement campaigns to GlobResp, but for other canopy components, will aid our understanding of the likelihood of respiration-induced biome changes. Such additional data will enable more rigorous benchmarking of different terrestrial model configurations of within-canopy respiration fluxes. Full mechanistic models, which can still be tested against GlobResp data, ultimately may allow further advances on empirical-based descriptions of respiration. However, availability of these remains a long way from routine usage, yet alone in large-scale climate models. This is an issue recently discussed in depth for the *b,c* instantaneous temperature response formulation^42, 43^, and where that exchange in the literature has relevance to more general respiration modelling.

## Methods

### Datasets

Two recently reported global datasets underpin Eq. (1). GlobResp describes patterns of temperature-normalised leaf respiration and associated leaf traits^5^. Respiration rates of sun-exposed leaves were measured for ~ 900 species, from 100 globally distributed sites covering from Arctic to the tropics. For each species, leaf respiration in darkness was measured during the day in situ on attached branches, or using detached branches that had their stems re-cut under water to maintain water flow to leaves. Leaves were dark-adapted for 30 min before each measurement, with respiratory CO_2_ release being measured using infra-red gas analyzers. Leaves were sampled, dried and analysed for total leaf nitrogen. The database^5^ shows that PFTs used by the JULES model capture much species variation across diverse sites. Respiration acclimates^16^ to prevailing ambient growth temperature, *T*_G_, and responses confirm (for identical temperatures) that cold-grown plants exhibit higher respiration rates than their warm-grown counterparts.^8, 9^

The second dataset describes variations in leaf respiration (in darkness) to instantaneous temperature changes^6^ based on 673 respiration-temperature curves from 231 species and 18 field sites. Leaves of detached branches of sun-exposed leaves were placed in a temperature-controlled cuvette and allowed to dark-adapt; leaf respiration was measured (using a Licor 6400 gas exchange system) as leaves were heated^6^ from 10 to 45 °C, at rate of 1 °C min^−1^. Convergence occurred in short-term temperature responses of *R*_d_ across biomes and PFTs; a model describing this *T*_l_- dependence is an exponential-of-quadratic.

### JULES modelling framework

The original JULES *R*_d_ description, with *Q*_10_ = 2.0, satisfies either *R*_d_ = *R*_d,25_ *Q*_10_^0.1(*T*^_l_^-25)^, or, with suppression at high and low temperatures, as (Eq. (18) of ref. ^2^) via additional denominator: *R*_d_ = *R*_d,25_ [*Q*_10_^0.1(*T*^_l_^-25)^] / [(1 + exp(0.3(13.0-*T*_l_))) × (1 + exp(0.3(*T*_l_-36.0)))]. This latter form is our Standard JULES simulations, and has similarities to others modelling respiration as linear in GPP, with GPP itself a peaked Arrhenius^44^ function of *T*_l_. The JULES value of *R*_d,25_ is linear in the maximum carboxylation rate of Rubisco at 25 °C, *V*_cmax,25_ (μmol CO_2_ m^−2^ s^−1^)^45, 46^. Parameter *f*_dr_ relates *V*_cmax,25_ to *R*_d,25_ as *R*_d,25_ = *f*_dr_ × *V*_cmax,25_, where *V*_cmax,25_ satisfies^47^ *V*_cmax,25_ = 10^6^ × *n*_e_ × *n*_l0_. Quantity *n*_l,0_ is the prescribed mass-based PFT leaf nitrogen concentration (kg N (kg C)^−1^) and *n*_e_ (mol CO_2_ m^−2^ s^−1^ kg C (k;gN)^−1^) links *V*_cmax,25_ to leaf nitrogen concentration. Table 1 shows how these equations and parameters give Standard JULES *R*_d,25_ values.

The parameters of Eq. (1) are given in Table 2, along with implication of GlobResp-based values^5^ for *R*_d,25_ values, when incorporated in to the JULES model.

The relative importance of contributions to revised *R*_d,25_ can be assessed from Tables 1 and 2. In general terms, and for broadleaf trees, the new representative *R*_d,25_ values change from 0.4157 to 1.136 μmol CO_2_ m^−2^ s^−1^. From the TRY database^18^, with 80% confidence, leaf nitrogen concentrations lie between 62% and 154% of their median value. This gives a range of 0.237 < *r*_1_ *n*_l,a_ < 0.593 μmol CO_2_ m^−2^ s^−1^. Growth temperature ranges of 5–25 °C give 0.2 < *r*_2_ *T*_G_ < 1.0 μmol CO_2_ m^−2^ s^−1^. This simple scale argument suggests a decreasing importance, both in terms of absolute and potential variability, of contributions to new *R*_d,25_ as new baseline, followed by acclimation and then by leaf nitrogen dependence.

Scaling to full canopy, respiration is modelled as declining exponentially in LAI above each point, *L*, as *R*_d_ exp(−*kL*) and *k* = 0.5. Hence all-canopy respiration *R*_d,c_ = *R*_d_ × [1−exp(−*kL*)]/*k*. This has modulation of *R*_d_ with light inhibition^21^, and any low soil moisture constraints^4^. Three additional components of respiration are those of roots, stems and growth. Root and stem respiration^4^ are linear in *R*_d,c_ (and thus *R*_d_) and dependent on estimated nitrogen concentrations in each. Canopy, root and stem respiration combine to an overall whole-plant maintenance respiration *R*_p,m_. Growth respiration, *R*_p,g_ is assumed to be a fixed fraction of GPP (Π_g_) minus *R*_p,m_ as *R*_p,g_ = *r*_g_ × [Π_g_ − *R*_p,m_], with coefficient *r*_g_ = 0.25. Whole plant respiration, *R*_p_, is the sum of maintenance *R*_p,m_ and growth *R*_p,g_ respiration, as *R*_p_ = *R*_p,g_ + *R*_p,m_.

JULES has a Dynamic Global Vegetation Model (DGVM) option, named Top-down Representation of Interactive Foliage and Flora Including Dynamics (TRIFFID)^4^, which uses calculated NPP (i.e. GPP-*R*_p_) to estimate LAI, in turn influencing PFT competition to derive their areal cover. This interactive vegetation model component enables, under major climate change, estimation of potential biome cover changes. To understand the implications of Eq. (1) on *R*_p_ without extra feedbacks via varying LAI, for this study we prescribe representative LAI and fractional covers for the PFTs at different geographical positions. This includes a prescribed fractional cover of agriculture, which is broadly representative of the current period, thus over-riding the TRIFFID component. However once additional data to refine within-canopy, root, stem and growth respiration estimates is available, building more confidence in total plant respiration estimates, then the TRIFFID component can be operated to assess future biome change likelihood. In the most general terms, the LAI and fractional cover of a PFT is strongly dependent on calculated NPP. If respiration increases significantly, this will lower NPP, lower LAI and reduce respiration (although it would also lower GPP).

### IMOGEN climate impacts modelling framework

The IMOGEN modelling system^23^ uses pattern-scaling to emulate GCMs. Radiative forcing *Q* (W m^−2^) is a single metric across greenhouse gases and aerosols that describes their overall influence on perturbed atmospheric energy exchanges. In IMOGEN, changes in *Q* drive a global energy balance model (EBM), which in turn multiplies patterns of climate change^22^. Such patterns are change in local and monthly meteorological conditions for each degree of global warming over land, with the latter estimated by the EBM. EBM parameterisation and patterns have been fitted against 34 GCMs in the Coupled Model Intercomparison Project Phase 5 (CMIP5) database^24^, including mapping from GCM native grids to 2.5° latitude × 3.75° longitude. Our climate change simulations are for historical, followed by atmospheric GHG concentrations of the RCP8.5 business-as-usual pathway^25^.

### Uncertainty analysis and site data

Uncertainty bounds^5^ on *r*_0_, *r*_1_ and *r*_2_ are repeated in Table 2. Naming these standard deviations as *ε*_0_, *ε*_1_ and *ε*_2_, then uncertainty on *R*_d,25_ for the New_*R*_d,25_ and New_*R*_d,25_ *_b,c* is $\sqrt {\varepsilon _0^2 + n_{\mathrm{l,a}}^2\varepsilon _1^2}$ whilst that for New_*R*_d,25_ *_b,c_*acclim is $\sqrt {\varepsilon _0^2 + n_{\mathrm{l,a}}^2\varepsilon _1^2 + T_{\mathrm{G}}^2\varepsilon _2^2}$. This therefore assumes that each of the three individual uncertainties are independent of each other. Bounds on parameter *b* and *c* in Eq. (1) are negligible^6^ and so multiplication by the exponent of Eq. (1) gives overall bounds on *R*_d_. This uncertainty is then passed through the JULES aggregation scheme in a similar way as that for the absolute dark respiration values, to give bounds on *R*_d,c_ and *R*_p_. Data and its related uncertainty bounds for Fig. 3 is from Manaus and Tapajos^48^, Caxiuana^49^, Mato Grosso^50^, Tambopata^51^, Iquitos^52^ and Guarayos^53^. Measurements a and b in locations Fig. 3f–h refer to different plots at the same location. At Tambopata, measurement a is for plot TAM05 and measurement b is for plot TAM06^51^; at Iquitos, measurement a is for plot Alp A and measurement b is for plot Alp C^52^; and at Guarayos, measurement a is for plot Kenia-dry and measurement b is for plot Kenia-wet^53^.

A review of the dependencies of ten other major land surface models shows that for six of these, upper canopy leaf respiration is dependent on leaf nitrogen content. The dependences are: BETHY is *V*_c,max_^54^; BIOME3 is *V*_c,max_^55^; BIOME-BGC is Nitrogen^56^; Century is Nitrogen^57^; CLM is Nitrogen^58^; LPJ is Nitrogen^28^; O-CN is Nitrogen^59^; Orchidee is Empirical^60^; Sheffield-DGVM is Nitrogen^61^ and TEM is Empirical^57^. The two models with dependence on *V*_c,max_ contain an implicit dependence on nitrogen, via assumed *V*_c,max_-N relationships.

In Fig. 5, we present data and model-based estimates of global NPP and GPP, divided into eight biomes that are in turn based on 13 in the WWF definitions of terrestrial ecoregions^36^. This reduction is by merging tropical, subtropical forests and mangroves into tropical forests; merging temperate mixed-forests and temperate conifers into (extratropical) mixed forests, and merging temperate grasses, flooded grasses and montane grasses into temperate grassland.

### Data availability

The GlobResp data is freely available from the TRY Plant Trait Database http://www.try-db.org/TryWeb/Home.php. The JULES model is freely available at http://jules.jchmr.org/. The code changes to the JULES respiration subroutine used in this analysis are available on request from Chris Huntingford (chg@ceh.ac.uk). All JULES model outputs, and for the four factorial experiments, are available for download from the Environmental Information Data Centre. The address is: https://doi.org/10.5285/24489399-5c99-4050-93ee-58ac4b09341a.

## Electronic supplementary material

Peer Review File
